# Genome Sequences of Three *Pseudoalteromonas* Strains (P1-8, P1-11, and P1-30), Isolated from the Marine Hydroid *Hydractinia echinata*

**DOI:** 10.1128/genomeA.01380-15

**Published:** 2015-12-10

**Authors:** Jonathan L. Klassen, Maja Rischer, Thomas Wolf, Huijuan Guo, Ekaterina Shelest, Jon Clardy, Christine Beemelmanns

**Affiliations:** a Department of Molecular and Cell Biology, University of Connecticut, Storrs, Connecticut, USA; b Leibniz Institute for Natural Product Research and Infection Biology e.V., Jena, Germany; c Department of Biological Chemistry and Molecular Pharmacology, Harvard Medical School, Boston, Massachusetts, USA

## Abstract

The genomes of three *Pseudoalteromonas* strains (P1-8, P1-11, and P1-30) were sequenced and assembled. These genomes will inform future study of the genes responsible for the production of biologically active compounds responsible for these strains’ antimicrobial, biofouling, and algicidal activities.

## GENOME ANNOUNCEMENT

Marine pseudoalteromonads are commonly associated with diverse marine eukaryotic hosts (1, 2) and exhibit a remarkable ability to produce small molecules with a broad range of bioactivities, including antibacterial (3), (anti)biofouling (4, 5), and algicidal (6) activities. We isolated three *Pseudoalteromonas* strains from the tissue of *Hydractinia echinata*, a colonial marine hydroid growing on gastropod shells inhabited by hermit crabs (*Pagurus pollicaris*). Sequencing these strains’ genomes will assist the manipulation of *Pseudoalteromonas* genomes, facilitate the discovery and production of new and biologically active molecules (7), and might provide insights into the molecular cues and mechanisms involved in the recruitment and settlement of *H. echinata* larvae (8).

Freshly collected *H. echinata* were purchased from the Marine Biological Laboratory (Woods Hole, MA, USA), and the tissue surface of feeding polyps were investigated for the presence of bacteria from the *Pseudoalteromonas* genus. Clean isolates were cultured in marine broth (Difco 2216) for 3 days at 30°C (150 rpm), and metabolites were extracted using standard solid-phase extraction methods. The resulting organic extracts were tested for antimicrobial activity against a broad range of human pathogenic bacteria and fungi, and showed weak to moderate antimicrobial activity against Gram-positive bacteria (e.g., *Staphylococcus aureus*). Genomic DNA was extracted using the GenElute blood genomic DNA kit (Sigma-Aldrich) according to the manufacturer’s protocol. Sequencing was performed at the Harvard Medical School Biopolymers Facility using Illumina TruSeq 50-bp paired-end libraries and a HiSeq2000 instrument (Illumina CASAVA version 1.8.2). A fraction of these reads representing ~50× coverage were assembled using the A5 pipeline version 201401013 (9) and screened for potential contaminations using blobology (10). Genomes were annotated using Prokka version 1.10 (11), and statistics were calculated using scripts from the Assemblathon 2 project (12).

The draft genome of strain P1-8 was sequenced to 50× coverage and comprises 37 contigs in 29 scaffolds, totaling 4,488,653 bases in length and having a G+C content of 41.2%. Its annotation includes 3,992 coding sequences (CDSs), 36 tRNAs, and 3 rRNAs.

The draft genome of strain P1-11 was sequenced to 51× coverage and comprises 44 contigs in 31 scaffolds, totaling 4,377,754 bases in length and having a G+C content of 41.0%. Its annotation includes 3,885 CDSs, 39 tRNAs, and 3 rRNAs.

The draft genome of strain P1-30 was sequenced to 51× coverage and comprises 51 contigs in 35 scaffolds, totaling 4,337,278 bases in length and having a G+C content of 40.9%. Its annotation includes 3,824 CDSs, 36 tRNAs, and 3 rRNAs.

Genes associated with biofilm formation and surface attachments, including genes encoding for *curli*, type II secretion system, type IV pili, and capsular polysaccharide (O-antigen) were identified, reflecting the adaptation to successful persistence and competition on marine surfaces (13). Genes encoding for secondary metabolite production (e.g., alterochromides), bacteriocins, and siderophore function (e.g., desferrioxamines) were detected using antiSMASH (14) and SMIPS (15). These genomes will promote the genetic analysis of the *Pseudoalteromonas* genus and will provide insights into secondary metabolite production and the molecular cues and mechanisms involved in the recruitment and settlement of *H. echinata* larvae (8).

### Nucleotide sequence accession numbers.

The whole-genome shotgun projects for strains P1-8, P1-11, and P1-30 have been deposited in DDBJ/EMBL/GenBank under the accession numbers LJSO00000000, LJSP00000000, and LKBC00000000, respectively. The versions described in this paper are the first versions, LJSO01000000, LISP01000000, and LKBC01000000.
